# Social media influence on Eating Disorders since COVID-19 pandemic: a pilot study

**DOI:** 10.1192/j.eurpsy.2023.509

**Published:** 2023-07-19

**Authors:** F. Micanti, G. Spennato, R. Claudio, E. Amoroso, M. D’Ambrosio, V. M. Saia, A. Barone, M. Tadic, D. Galletta, M. Vannini

**Affiliations:** Psychiatry and Psychology, “Federico II” University Medical School, Naples, Italy

## Abstract

**Introduction:**

Several studies show a negative impact of mass media contents on adolescents’ mental health, especially on perceived body uneasiness. COVID-19 lockdown determined an increased use of social networks (SN). Psychiatrists highlighted an increase in Eating Disorders’ (ED) diagnoses.

**Objectives:**

The aim of this study is to assess the pattern of SN use in patients with ED using a self-administered questionnaire.

**Methods:**

30 patients with clinical diagnosis of ED (Anorexia nervosa, Bulimia nervosa or Binge eating disorder) admitted to the ED unit, underwent clinical assessment, and filed a questionnaire on SN use. The questionnaire assesses time spent on SN, weight-control apps use, exposure to fitness- or food-related contents and to ED-promoting contents, distractibility, weight changes and feeling of body uneasiness.

**Results:**

Mean age was 20.63 (SD 4.71), mean BMI 20.24 (SD 5.27); 93.3% (28) of patients were females. Eating behaviours were divided into restrictive type (66.7%, 20) or binge/bulimic (33.3%, 10). 16.7% (5) of patients reported self-injury behaviours. In 46.7% (14) of cases, the onset of the ED occurred during COVID-19 pandemic; the remaining 53.3% (16) experienced a relapse of a previous ED during this period. 66.7% (20) of subjects reported an increased use of social media and fitness apps. 90% (27) experienced weight changes during the pandemic, with 76.7% (23) seeking nutritional or psychological interventions. 53.3% (16) perceived an increase in body- or food-related contents on their SN feeds, with 50% declaring of knowing the meaning of the terms pro-ana and pro-mia.

Table 1 displays reported answers to the questionnaire. Table 2 shows mean age of patients according to self-injury behaviours and to the onset time of ED.

**Table 1.** 

**Questionnaire subscales (n)**	**Rarely occurred % (n)**	**Often occurred % (n)**
Increase of time spent on SNs (30)	33.3% (10)	66.7% (20)
Distractibility (30)	70% (21)	30% (9)
Self-injury contents (30)	96.7% (1)	3.3% (29)
Body uneasiness (30)	26.7% (8)	73.3% (22)
Pro-ana/pro-mia contents influence (15)	53.3% (8)	46.7% (7)
Body- and food-related contents influence (30)	20% (6)	80% (24)

**Table 2.** 

**Eating disorders features (n)**	**Mean age (SD)**	
Self-injury – Yes (5)	18.20 (1.92)	p<0.05
Self-injury – No (25)	21.12 (4.97)	
Onset during Covid-19 pandemic (14)	18.29 (1.82)	p<0.01
Worsening during Covid-19 pandemic (16)	22.69 (5.51)

**Conclusions:**

ED onset during the COVID-19 pandemic and self-injury behaviours appear as pivotal characteristics of younger patients, displaying a greater severity of the disorder in our clinical experience. With a more consistent number of patients, it would be possible to correlate SN use and body- and food-related contents to the onset and the severity of ED, focusing on pandemic periods.

**Disclosure of Interest:**

None Declared

